# The complete chloroplast genome sequence of *Abies chensiensis* (Pinaceae: Abietoideae), an endangered species endemic to China

**DOI:** 10.1080/23802359.2018.1507636

**Published:** 2018-10-15

**Authors:** Weimin Li, Chen Chen, Guoqing Bai, Bin Li, Hao Chen, Yafu Zhou, Sifeng Li

**Affiliations:** a Xi’an Botanical Garden of Shaanxi Province (Institute of Botany of Shaanxi Province), Xi’an, China;; b Shaanxi Province Qinling- Bashan Mountains Engineering Research Center of Conservation and Utilization of Biological Resources, Xi’an, China

**Keywords:** *Abies chensiensis*, Illumina sequencing, chloroplast genome, MITObim

## Abstract

Chloroplast (cp) genome sequences became a widely used tool for evolutionary and phylogenetic studies in plants. *Abies chensiensis* (Pinaceae: Abietoideae) is an endangered species endemic to China. To understand its evolutionary characteristics, the complete chloroplast genome of the *A. chensiensis* has been reconstructed from the whole-genome Illumina sequencing data. The circular genome is 121,784 bp in length and without a typical quadripartite structure due to the loss of IR region. The total GC content of whole genome sequence is 38.3%. The chloroplast genome encodes 109 genes, including 75 protein-coding genes, 30 transfer RNA genes and four ribosomal RNA genes. Among them, 35 genes involved in photosynthesis, while 58 genes involved in self-replication. The Maximum-Likelihood phylogenetic analysis showed a strong sister relationship with *A. nephrolepis and A. koreana* in Abietoideae. Our findings provide fundamental information for further evolutionary and phylogenetic researches of Abietoideae.

*Abies* is an important genus of the Pinaceae family, and *Abies chensiensis* is endemic to China and has been listed as critically endangered species in China Plant Red Data Book as one of the II class conservation plants (Liu and Zhang 2003). *Abies chensiensis* distributed in Qinling and Bashan mountains with altitude from 1800–3500 m. Complete chloroplast genome sequences in *Abies* such as *Abies koreana* and *Abies nephrolepis*, have been reported (Yi et al. 2015; Yi et al. 2016). Therefore, sequencing and analysis of chloroplast genome structure of *A. chensiensis* will contribute to a better understanding of the evolutionary mode of the chloroplast genome and provide essential genomic information for protection of the critically endangered species.

The complete chloroplast genome of *A. chensiensis* is a circular DNA molecule with 121,784 bp in length and was deposited in GenBank with the accession number MH510244. The overall GC content of the cp genome is 38.3% GC content. Compared with other higher plants, chloroplast genomes of *A. chensiensis* has no typical quadripartite structure due to the loss of IR region, which is common in Abietoideae (Zhang et al. 2006). This chloroplast genome encodes 109 functional genes, including 75 protein-coding genes (PCGs), 30 tRNA genes and four rRNA genes. Among them, 35 are involved in photosynthesis, and 58 genes are involved in self replication. All genes are single copy while *ycf*12 and *rps*12 are double copy in genome. Moreover among all the protein-coding genes, three genes (*atp*F, *rpl*2 and *rpoC*1) have one intron, and gene *ycf*3 harbors two introns, while other 71 genes are intronless. Fifty-four genes locate in the forward strand and 55 genes locate in the reverse strand.

Fifty-seven conserved PCGs sequences from 14 chloroplast genomes were aligned by MAFFT (Katoh et al. 2002) and then were connected as gene strings. The Maximum-Likelihood phylogenetic tree of *F. dibotrys* was generated using those gene strings sequence by MEGA 6.0 (Tamura et al. 2013) with using 500 bootstrap replicates (Figure 1). The Maximum-Likelihood phylogenetic analysis showed a strong sister relationship with *A. nephrolepis and A. koreana* in Abietoideae. Our findings provide fundamental information for further evolutionary and phylogenetic researches of Abietoideae.

**Figure 1.**
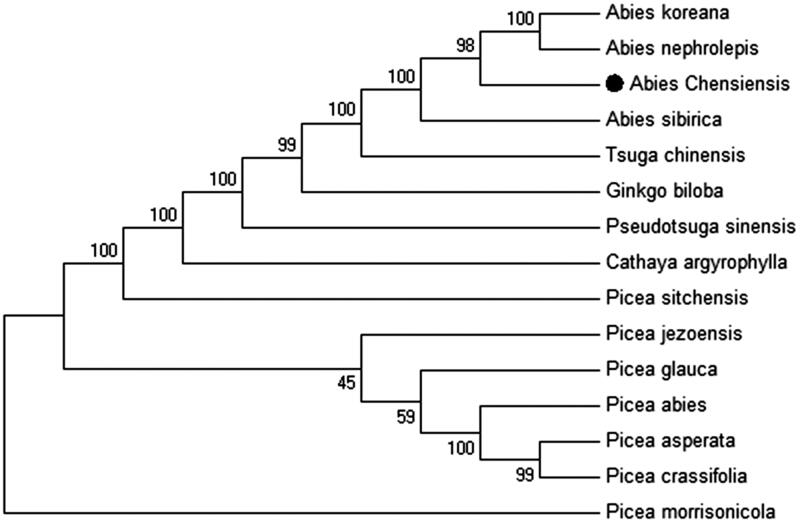
Phylogenetic of 14 species based on the Maximum-Likelihood analysis of the whole chloroplast genome sequences using 500 bootstrap replicates. The analyzed species and corresponding Genbank accession numbers are as follows: *Abies nephrolepis* (KT834974.1), *Abies koreana* (KP742350.1), *Abies sibirica* (KR476376.1), *Cathaya argyrophylla* (AB547400.1), *Ginkgo biloba* (JN654343.1), *Picea abies* (HF937082.1), *Picea asperata* (KY204451.1), *Picea crassifolia* (KY204450.1), *Picea glauca* (KT634228.1), *Picea jezoensis* (KT337318.1), *Picea morrisonicola* (AB480556.1), *Picea sitchensis* (EU998739.3), *Pseudotsuga sinensis* (AB601120.1), *Tsuga chinensis* (LC095866.1).
